# PDMS Sponges with Embedded Carbon Nanotubes as Piezoresistive Sensors for Human Motion Detection

**DOI:** 10.3390/nano11071740

**Published:** 2021-07-01

**Authors:** Blake Herren, Vincent Webster, Eric Davidson, Mrinal C. Saha, M. Cengiz Altan, Yingtao Liu

**Affiliations:** School of Aerospace and Mechanical Engineering, University of Oklahoma, Norman, OK 73019, USA; blake.herren@ou.edu (B.H.); Vincent.G.Webster-1@ou.edu (V.W.); ericdavidson@ou.edu (E.D.); msaha@ou.edu (M.C.S.); altan@ou.edu (M.C.A.)

**Keywords:** piezoresistive sensor, wearable electronics, nanocomposite sponge, human motion monitoring, semiconductors, carbon nanotubes, elastomers, flexible sensors

## Abstract

Porous piezoresistive sensors offer promising flexible sensing functionality, such as human joint motion detection and gesture identification. Herein, a facile fabrication method is developed using a microwave-based rapid porogen removal technique for the manufacturing of porous nanocomposite sponges consisting of polydimethylsiloxane (PDMS) and well-dispersed carbon nanotubes (CNTs). The porogen amounts and CNT loadings are varied to tailor the porosity and electrical properties of the porous sensors. The sponges are characterized by a scanning electron microscope (SEM) to compare their microstructures, validate the high-quality CNT dispersion, and confirm the successful nanofiller embedding within the elastomeric matrix. Sponges with a 3 wt% CNT loading demonstrate the highest piezoresistive sensitivity. Experimental characterization shows that the sponges with low porosity have long durability and minimal strain rate dependence. Additionally, the developed sponges with 3 wt% CNTs are employed for the human motion detection using piezoresistive method. One experiment includes fingertip compression measurements on a prosthetic hand. Moreover, the sensors are attached to the chest, elbow, and knee of a user to detect breathing, running, walking, joint bending, and throwing motions.

## 1. Introduction

Many fabrication methods have been developed recently for a wide variety of flexible sensors in several applications including human motion detection [1], sensor arrays [2], soft robotics [3], biomechanics [4], structural health monitoring [5], and prosthetic devices [6]. These sensors often consist of an elastic polymer that provides the flexible backbone of the sensor while metallic or carbonaceous nanoparticles provide electrical conductivity to the nanocomposites. Common nanoparticles used to improve conductivity in flexible sensors include gold and silver nanowires [7], graphene [8], carbon black [9], and carbon nanotubes (CNTs) [10,11]. These highly flexible nanocomposites have been used to produce a signal in response to mechanical input inducing a change in the measurable electrical properties of the material. Three sensing mechanisms are typically demonstrated in the field including capacitive [12], piezoelectric [13], and piezoresistive effects [14]. Piezoresistivity is more commonly implemented when developing these sensors due to facile signal collection and fabrication. Highly flexible piezoresistive pressure sensors typically decrease their electrical resistance under compressive strain mainly due to movement of conductive nanoparticles forming new conductive networks. Additional mechanisms have been developed and employed to improve the sensitivity of flexible piezoresistive sensors by implementing SiO_2_ microparticles to break apart conductive networks [15], microcracks on a highly conductive surface [16], and porosity [17]. Particularly, introducing porosity in flexible sensors has received considerable attention due to facile fabrication methods, enhanced compressibility and sensitivity, and increased commercial applications [18]. 

Numerous foams and sponges have been fabricated using different materials to develop highly compressible piezoresistive sensors. Often, the multi-functionality of the flexible sponges is introduced through the dispersion of nanoparticles within the elastomeric matrix or attaching the nanoparticles to the pore walls. Some of the most common piezoresistive sponge fabrication techniques include dip-coating a neat polymer sponge in a suspension of dispersed conductive nanoparticles [19,20,21], sugar templating method where a sugar cube is infiltrated with resin and used as a sacrificial porogen [20,22,23,24], and mixing salt or sugar porogen with the nanocomposite prepolymer before curing [25,26]. Sponges made of polydimethylsiloxane (PDMS) have received significant attention for their biocompatibility and wide functionality including triboelectric generators [27], oil/water separation [28], and piezoresistive sensors [29]. 

Piezoresistive PDMS sponges have been recently developed for applications including detecting speaking, breathing, and many activities requiring large human motions [29]. These sensors are often applied to the skin of a user which requires the sensor to be biologically safe for the individual to have close contact [30]. Piezoresistive sponges with nanoparticles on the walls of the sensor have a high likelihood of nanoparticle detachment under dynamic loading which could prove to be harmful to the users and likely detrimental to the durability of the sensor [20]. In contrast, when nanoparticles are fully embedded within the PDMS matrix, there is a significantly lower likelihood of nanoparticle detachment from the sensor during use. Thus, the sensor can be safely applied on the skin of a user for motion detection applications. 

Removing sacrificial porogens like salt or sugar from PDMS nanocomposites during sponge fabrication is a time-consuming process. It has been reported that it could take many hours or even days to fully remove porogens by soaking cured nanocomposites in a warm solvent. New fabrication methods are necessary to reduce processing time for rapid manufacturing of porous and flexible functional nanocomposites. To solve these problems, one potential solution is to implement microwave processing techniques during nanocomposite fabrication. In our previous studies, microwave irradiation was utilized to rapidly cure PDMS/CNT nanocomposites in 25 s or less due to the strong microwave absorption of CNTs [17]. The major findings from these microwave-curing studies included significantly enhanced electrical conductivity [31], decreased compressive modulus, and unaffected piezoresistive sensitivity of the nanocomposite sensors [32]. During these studies, we observed the nanocomposite material expanded significantly while under microwave irradiation due to thermal shock within the viscoelastic matrix. 

Herein, PDMS sponges containing dispersed CNTs were developed for human motion detection applications. A novel microwave irradiation method was used to rapidly remove the sugar porogens of a fully cured heterogeneous mixture of sugar and PDMS containing embedded CNTs. During the microwave-assisted porogen removal, the fully cured CNT-based nanocomposite absorbed significant heat and expanded the sponge walls to allow water to easily flow through the continuous open-cell pores and dissolve the sugar rapidly. The electrical, piezoresistive, and mechanical properties of the sensors were tailored by adjusting the CNT loading and sugar porogen amount. Scanning electron microscopy (SEM) was employed to image the microstructure, the quality of nanofiller dispersion, and to confirm the CNTs were embedded within the polymer. The sensor properties were fully characterized, and various sensing applications on a 3D printed prosthetic hand and skin-attachable human motion detection were demonstrated.

## 2. Materials and Methods

### 2.1. Materials

Multi-walled CNTs, with an average diameter between 50–80 nm and aspect ratio >100, were purchased from Sigma-Aldrich, USA. Tetrahydrofuran (THF) was purchased from Sigma-Aldrich, USA. The SYLGARD 184 PDMS kit was purchased from Dow Corning, USA. Cane sugar was purchased from Walmart. Unless otherwise noted, all materials were used as received and as the manufacturer recommended. 

### 2.2. Nanocomposite Sponge Fabrication

**Figure 1:**
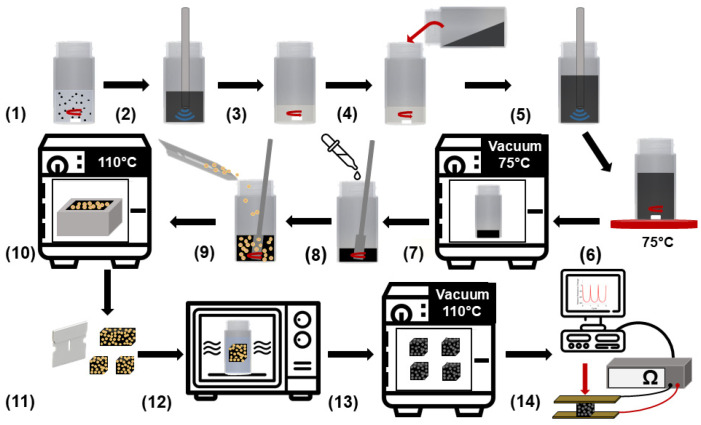
Schematic of the complete fabrication method used to manufacture the porous nanocomposite sensors. The steps include: (**1**) CNTs mixed in THF, (**2**) suspension sonicated, (**3**) PDMS and THF mixed, (**4**) CNT suspension poured into PDMS and THF solution, (**5**) mixture sonicated, (**6**) mixture stirred and THF evaporated on a hot plate, (**7**) resin degassed in a vacuum oven, (**8**) PDMS curing agent added to nanocomposite resin, (**9**) prepolymer mixed with sugar, (**10**) nanocomposite cured in an oven, (**11**) cube samples cutout of cured nanocomposite, (**12**) microwave-assisted porogen removal, (**13**) samples dried in a vacuum oven, and (**14**) sponges tested as piezoresistive sensors.

The nanocomposite sponge sensors were fabricated with a solvent-based sonication method to disperse the CNTs well within the PDMS matrix. The fabrication method used in this study can be seen in Figure 1. First, CNTs were measured and mixed in 50 mL of THF with a magnetic stir bar. The CNT suspension was then ultrasonicated with a probe tip sonicator for 10 min, pulsing on for 5 s and off for 2 s. During the sonication process, PDMS Part A was measured and mixed in THF with a magnetic stir bar. After sonication, the dispersed CNT suspension was poured into the PDMS/THF solution, mixed with a magnetic stir bar for 2 min, and sonicated for 30 min. Next, the mixture was placed on a 75 °C hot plate and mixed until the magnetic stir bar stopped spinning due to THF evaporation. Then, to fully evaporate the THF from the nanocomposite resin, the material was held under vacuum at 75 °C overnight. Next, the curing agent was added at a 10:1 ratio (Part A: Part B) and mixed by hand to create the prepolymer (PDMS/CNT). A predetermined amount of sugar porogen was added to PDMS/CNT and mixed by hand. The moldable nanocomposite mixture was cured in an oven at 110 °C and a razor blade was used to cut out cube-shaped sensors of roughly 10 × 10 × 10 mm. Nanocomposite sponges were prepared at a constant sugar porogen amount of 70 wt% with a varying CNT loading including 1.5 wt% (CNT1.5P70), 2 wt% (CNT2P70), 2.5 wt% (CNT2.5P70), and 3 wt% (CNT3P70). Additionally, sponges were fabricated at a constant CNT loading of 3 wt% and varying sugar porogen amounts including 75 wt% (CNT3P75), 80 wt% (CNT3P80), and 85 wt% (CNT3P85). Designations for the sponge materials fabricated in this study are also shown in Table S1.

### 2.3. Rapid Porogen Removal

To rapidly remove the sugar porogen from the cured nanocomposite, a novel microwave irradiation leaching process was used. Each sample was placed in a glass bottle with 100 mL of distilled water and heated under microwave irradiation for several pulses, replacing the water after each pulse. A General Electric 1200-watt unmodified commercial microwave oven was used for the porogen removal process. The CNT1.5P70, CNT2P70, CNT2.5P70, and CNT3P70 nanocomposite sponges were pulsed 5 times in the microwave for 60 s, 55 s, 50 s, and 45 s, respectively. The CNT3P75, CNT3P80, and CNT3P85 sponges were pulsed 5 times in the microwave for 43 s, 40 s, and 37 s, respectively. After complete removal of the sugar porogen, the samples were dried in a vacuum oven overnight. To quantify the rate of porogen removal, fully cured 3 wt% nanocomposites containing 70 wt% sugar were weighed between each microwave pulse after drying the samples completely. 

### 2.4. SEM

Before imaging with a Zeiss Neon 40EsB SEM, each sample was sputter-coated with approximately 10 nm of AuPd to diminish charging artifacts. The pores of each sample containing a constant CNT loading (3 wt%) and various loadings of sugar porogen (70 wt%, 75 wt%, 80 wt%, and 85 wt%) were imaged to compare the microstructure of the sponges. In addition, CNTs were imaged on the cut surface of the sponge to confirm a high-quality nanoparticle dispersion was achieved within the elastomeric matrix, and an uncut surface was imaged to show the quality of CNT embedding in the matrix.

### 2.5. Piezoresistive Sensor Characterization

To investigate and compare the performance of each sensor fabricated, dynamic compression tests were performed on an Instron 3345 single column mechanical testing machine with a 100 N load cell. The piezoresistive sensors were placed in between two copper plates that were electrically connected to a Hioki RM3545-02 Resistance Meter to measure the resistance of the semi-conductive sensor throughout each dynamic compression test. Using this experimental setup, the sensors were cyclically compressed for 10 cycles at each maximum strain including 2%, 3%, 5%, 10%, 25%, and 50% at a constant strain rate of 2%/s. The mechanical properties including the stress–strain curves and compressive moduli were determined from the first cycle of 50% maximum strain. The piezoresistive functionality including the durability and strain rate dependence of the CNT3P70 and CNT3P85 were compared. The durability test included 360 cycles of 50% maximum compressive strain at a constant strain rate of 50%/min. The varying strain rate test included 10 cycles at each strain rate (20%/min, 100%/min, 250%/min, 500%/min, and 1000%/min) for each compressive strain (5%, 10%, 25%, and 50%) applied. 

### 2.6. Flexible Sensor Applications

The piezoresistive sensors were attached to the fingertips of a 3D printed prosthetic hand with PDMS sleeves and copper tape electrodes. The sensors demonstrated a change in resistance due to the pressure-induced on the fingertips of the prosthetic while cyclically grabbing an object. Additionally, the sensors were taped on the chest, inside of the elbow, and behind the knee of a user. The user compared basic human motions including breathing slow versus fast, walking versus running, and elbow bending versus a throwing motion to demonstrate the ability of the sensor to detect various human motions. 

## 3. Results and Discussions

### 3.1. Rapid Porogen Removal

**Figure 2:**
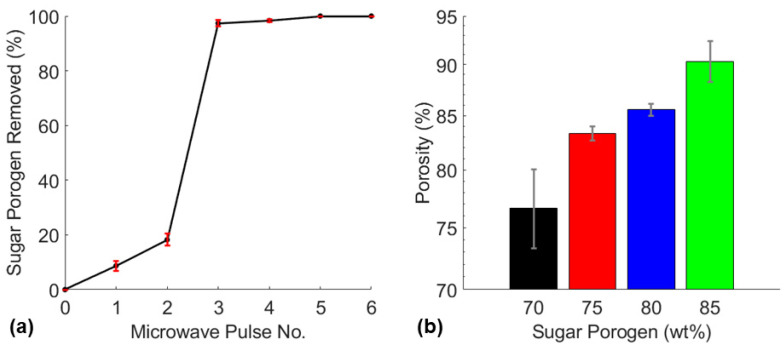
(**a**) Sugar porogen removed after each microwave pulse of CNT3P70 sponges and (**b**) the varying porosities of CNT3P70, CNT3P75, CNT3P80, and CNT3P85 sponges.

The ability to control the porosity of a piezoresistive sponge is an important capability for the fabrication that most methods lack. Porosity has a major impact on the mechanical and piezoresistive properties of these porous sensors. Porous sensor manufacturing methods should have the ability to control the porosity of the sensors as the compressibility of these sensors may greatly influence the appropriate applications, pressure sensitivity, and their durability. The fabrication method developed in this study allows the manufacturer to significantly vary the porosity and compressive modulus of the sensors by varying the sugar porogen amount between 70 wt%–85 wt%. The varying porosity measurements of the fabricated sensors and the percentage of sugar porogen removed for each microwave pulse are shown in Figure 2.

The quantification of sugar porogen removal during the microwave-based leaching process revealed the sugar porogen was 100% removed after the fifth pulse (Figure 2a). Additionally, the experiment showed that more than 80% of the total sugar porogen was removed after the third microwave pulse. This may likely be explained by the fully cured sugar infiltrated nanocomposite being unable to expand under microwave irradiation in the first two pulses due to the sugar template constricting the elastomer. However, before the third pulse, almost 20% of the sugar porogen had been removed which allowed the sponge to expand and open its pores leading to significant porogen dissolution due to microwave-induced thermal expansion. The last two pulses removed residual sugar that remained on the pore walls of the sponge. The fabrication technique demonstrated the ability to remove sugar porogen amounts of 70 wt%, 75 wt%, 80 wt%, and 85 wt% which produced nanocomposite sponges with porosities of 77%, 83%, 86%, and 90%, respectively (Figure 2b).

Many studies have used a variety of materials as a porogen to create PDMS sponges and several mentioned the difficulty and time-consuming process typically required to remove the porogen. Table 1 shows the time required to remove the porogen in this study versus other studies that reported this time. Nanoparticles in parenthesis were attached to the surface of a neat PDMS sponge while nanofiller listed after a slash were dispersed within the PDMS matrix. This is noteworthy as typically the stiffness of an elastomer is increased with nanofiller making porogen removal more difficult. However, this study used the embedded CNTs to aid in the porogen removal process, resulting in a process that required less than 5 min to complete. This impressive time efficiency demonstrated the potential for this method to be valuable for the mass production of these nanocomposite sponges.

### 3.2. Sponge Structure

**Figure 3:**
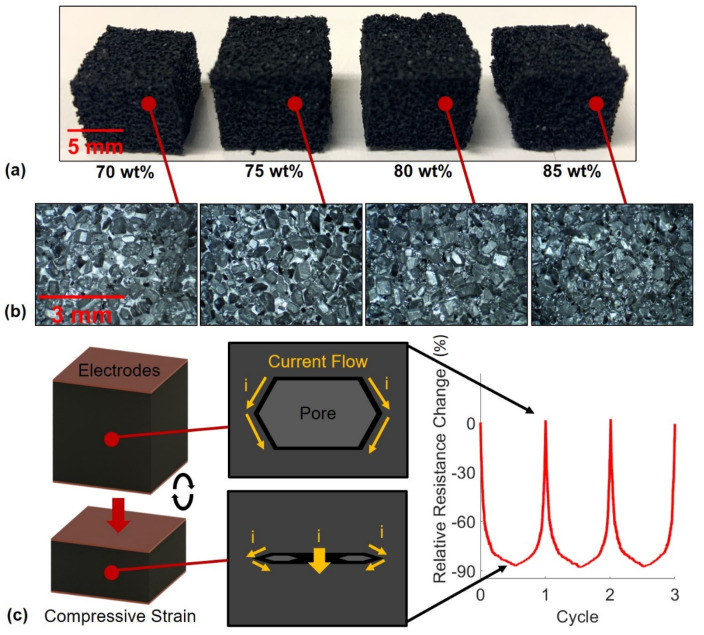
(**a**) Various porosity sponges with CNT loadings of 3 wt%, (**b**) microstructure of the varying porosity sponges, and (**c**) schematic illustrating the piezoresistive mechanism of a collapsed pore in the sponge sensors.

The goal of developing these flexible nanocomposite sensors was to make a device that was highly porous for facile compressibility and safe for use on the human body. Several studies in the field have used a simple dip-coating method to attach nanoparticles to the surface of the foam or sponge samples to functionalize the material [19,20,21]. However, many of these studies ignored the high likelihood of nanoparticle detachment. Detachment of CNTs from a sponge during use as a skin-attachable human motion detection device may introduce serious health risks to the users [30,43]. In this study, CNTs were fully dispersed within the PDMS matrix to significantly reduce the risk of nanoparticle detachment. Therefore, the piezoresistive behavior of the sensors may largely be attributed to the collapsing of semi-conductive pores within the samples during compression as illustrated in Figure 3. Additional piezoresistive mechanisms in the compression sensors may include surface effects of the contact between the elastomeric nanocomposite and copper electrodes and a reduction in average tunneling distance as conductive nanofiller are brought closer together within the matrix.

**Figure 4:**
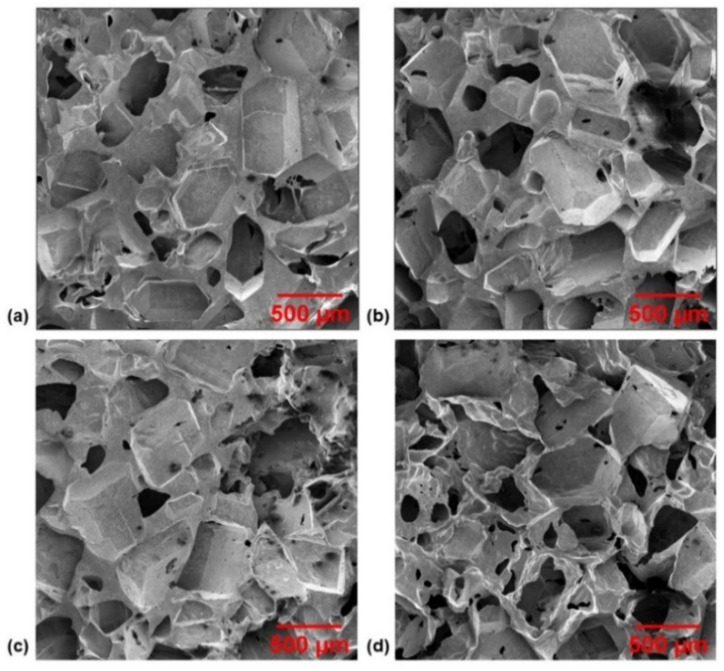
SEM images of the microstructure of various porosity sponges with 3 wt% CNT loadings and (**a**) 70 wt%, (**b**) 75 wt%, (**c**) 80 wt%, and (**d**) 85 wt% sugar porogen.

**Figure 5:**
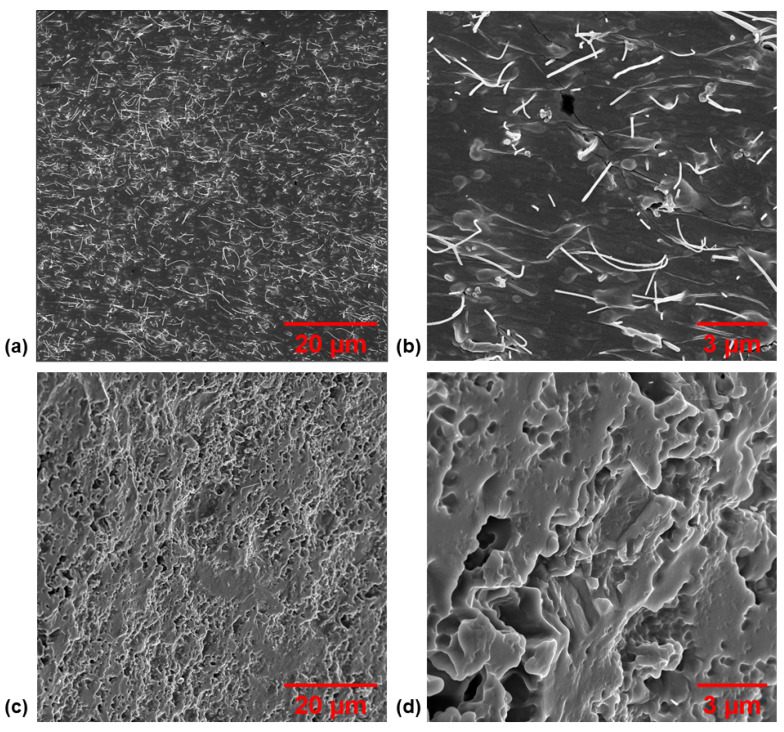
SEM images of the nanostructure of a nanocomposite sponge including (**a**,**b**) a cut surface of sponge exposing well-dispersed embedded CNTs and (**c**,**d**) an uncut surface of a sponge demonstrating successful embedding of the nanofiller within the polymer matrix.

To visually investigate the structure of the sponges including the varying porosity, quality of CNT dispersion within the matrix, and determine if CNTs were successfully embedded into the polymer, SEM images were taken. First, the microstructures of the varying porosity sponges were imaged and the results for CNT3P70, CNT3P75, CNT3P80, and CNT3P85 are shown in Figure 4. As expected, a higher porosity sponge displayed a greater number of pores visible in the microstructure. These pores were a pathway for water to travel during the porogen removal process, which explains why removing sugar was easier and required shorter microwave pulse times for samples with a higher amount of sugar porogen. Next, the nanostructure of a sponge was imaged, and the results are shown in Figure 5. The surface that was cut with a razor blade was imaged to determine the quality of CNT dispersion within the matrix. No noticeable agglomerates were seen during imaging and the images shown in Figure 5a,b demonstrated a uniform distribution of CNTs within the PDMS matrix. The uncut surface of the sponge was also imaged to confirm the successful embedding of the CNTs within the polymer matrix (Figure 5c,d). 

### 3.3. Piezoresistive Sensor Characterization

**Figure 6:**
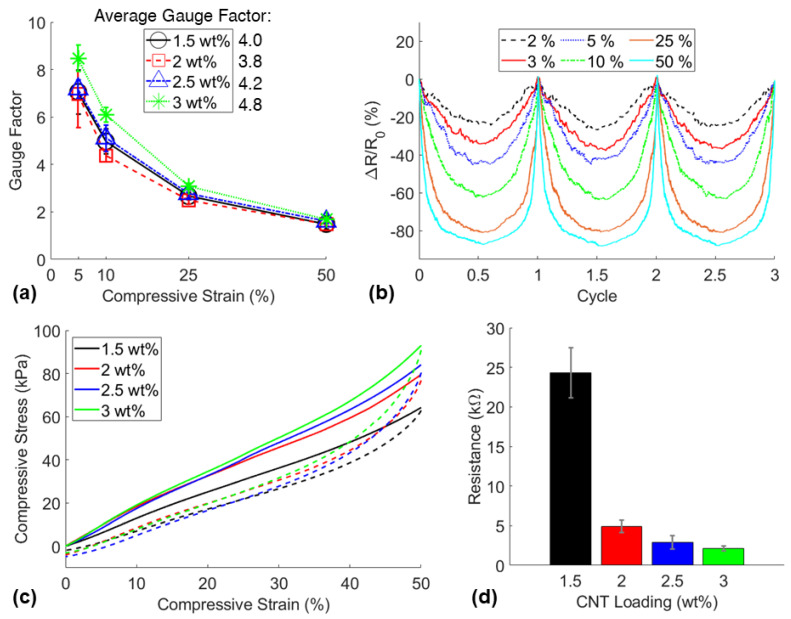
(**a**) Sensitivity comparison of CNT1.5P70, CNT2P70, CNT2.5P70, and CNT3P70 sponges, (**b**) representative relative resistance change signals of a CNT3P70 sponge under maximum compressive strains of 2%, 3%, 5%, 10%, 25%, 50%, respectively, (**c**) stress–strain curves of sensors in compression, and (**d**) the average resistances of the sensors.

The fabrication technique implemented in this study gives the ability to fabricate piezoresistive sponges with varying porosity and varying CNT loading. Therefore, it was necessary to characterize and compare the piezoresistive sensitivity of the sensors fabricated with various formulations to determine the differences in behavior, benefits, and potential applications of different sponge sensors. Notably, the sensitivity in a wide strain range of every fabricated sensor was compared by determining the gauge factor at each applied strain. The gauge factor was calculated using Equation (1).

(1) $$Gauge\;Factor=\frac{R-R_{0}}{R_{0}\;\cdot \left(\varepsilon -\varepsilon_{0}\right)}=\frac{∆R}{R_{0}\;\cdot \;∆\varepsilon }$$

where *R* designates the immediate resistance of the sensor, *R*_0_ designates the initial resistance, ε is the maximum strain applied, and ε_0_ is the preloaded strain. In this study, the preloaded strain was kept constant while the sensors were cyclically loaded at varying maximum compressive strains between 1–50%. The piezoresistive signals and the mechanical properties of each signal were determined by these compression tests. The results of the comparison of CNT1.5P70, CNT2P70, CNT2.5P70, and CNT3P70 sponges are shown in Figure 6. 

Although similar piezoresistive sensitivity was observed in each sensor, the sponges containing 3 wt% CNT loading demonstrated the highest average sensitivity with a gauge factor of 4.8 (Figure 6a). Additionally, the CNT3P70 sensors displayed the best minimum compressive strain detection of 2% (Figure 3b). The mechanical properties were not significantly affected by the varied loading of CNTs with a relatively consistent compressive modulus of 160 ± 29 kPa between all the sensors. No significant trend was found for the compressive modulus or porosity of each sensor, likely due to significant variations in viscosity between the nanocomposite resins [17], variations in sugar particle sizes, and potential inhomogeneity during the mixing, resulting in spatial porosity variations in the samples (Figure S1). Representative stress–strain curves for the sponges with varied CNT loadings and consistent sugar porogen loading of 70 wt% are shown in Figure 6c. The electrical properties were significantly impacted by the nanofiller loading as the measured resistance was an order of magnitude different between CNT loadings of 1.5 wt% and 3 wt%. The highest CNT loading (3 wt%) displayed the highest number of conductive networks, and therefore the lowest resistance, which proved to be beneficial for establishing reliable contacts with the copper electrodes. Due to demonstrating the highest piezoresistive sensitivity and the highest electrical conductivity, 3 wt% CNT loading was chosen to fabricate sensors with various sugar porogen amounts.

To evaluate the sensor performance, the gauge factor of the developed nanocomposite sensors was compared with those published in the literature (Table 2). Since multiple factors, including nanoparticle type, applied strain, porosity, nanoparticle concentration, applied strain rate can all impact the measured gauge factor, the gauge factor of this work was only compared to PDMS based nanocomposites reinforced by carbon-based nanoparticles. When the applied strain is in the 10–15% range, the gauge factor reported in this work is comparable to other published nanocomposite sensors. 

**Figure 7:**
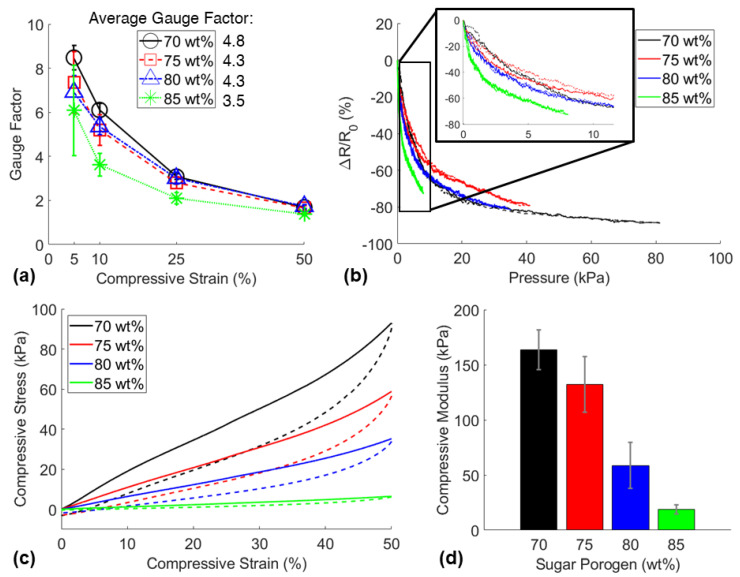
(**a**) Piezoresistive sensitivity comparison of sponges fabricated with various sugar porogen loadings and 3 wt% CNT loading, (**b**) representative relative resistance change signals of each sensor due to pressure applied during a 50% strain cycle, (**c**) stress-strain curves of sensors up to 50% compression, and (**d**) compressive modulus of the sensors.

Sugar porogen content was varied to demonstrate the wide range of mechanical behavior of the sponges. Additionally, the piezoresistive sensitivities of the sponges with various porosities were compared to determine the best performing sensor with the highest gauge factor. The results of this study are shown in Figure 7.

The lowest porosity sensor displayed the highest average gauge factor between 5–50% strain, likely due to the pores within the more porous sponges not collapsing fully within this strain range (Figure 7a). However, the most porous sensors fabricated with 85 wt% porogen were more sensitive to applied pressure as shown in Figure 7b. This was due to the significantly lower compressive stiffness of the more porous sponges as the average compressive moduli of sensors fabricated with 70 wt%, 75 wt%, 80 wt%, and 85 wt% were 164 kPa, 132 kPa, 59 kPa, and 18 kPa respectively (Figure 7d). The stress-strain curves in Figure 7c denote typical viscoelastic behavior where the area between the loading (solid line) and unloading (dashed line) curves is the energy absorbed by the material and the area below the unloading curve is the energy recovered. Clearly, the lower porosity sponges displayed greater energy absorption and energy recovery than the higher porosity sponges. This proves the key capability of the developed manufacturing method to fabricate piezoresistive sponges with controllable mechanical response, offering the potential for the sensors to be used in a wide variety of applications. Further comparison of the piezoresistive function for the highest porosity sponge (CNT3P85) and the lowest porosity sponge (CNT3P70) was explored to determine the differences in piezoresistive behavior for different porosity sponges. 

**Figure 8:**
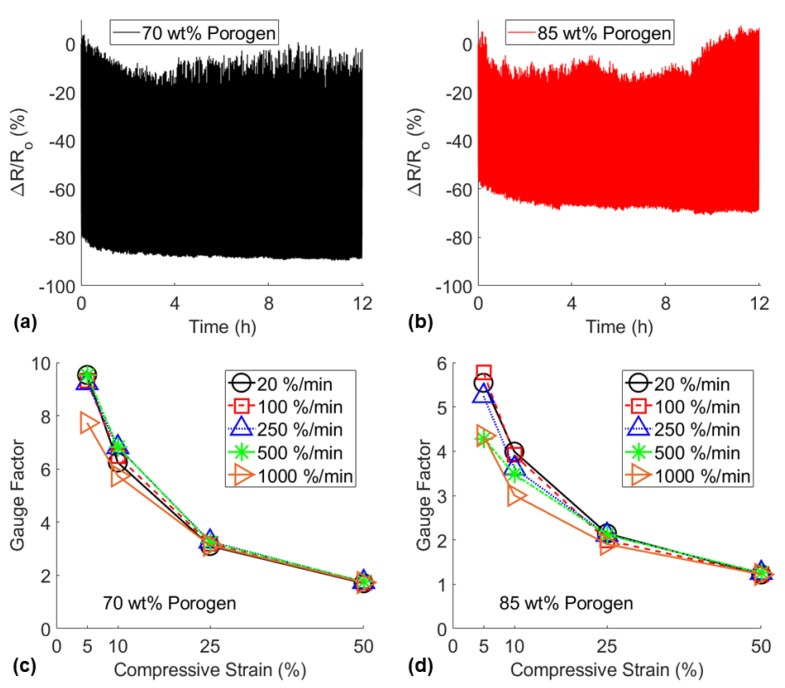
Piezoresistive signals for the 360-cycle durability test to compare (**a**) CNT3P70 and (**b**) CNT3P85 sensors and gauge factors at each compressive strain for varying strain rates (20 %/min–1000 %/min) to compare (**c**) CNT3P70 and (**d**) CNT3P85 sensors.

To determine the usability of these sensors, long-term durability and strain rate dependency tests were performed, in addition to step-sensing and viscoelastic creep relaxation tests that are shown in Figures S2 and S3. The results of the durability and strain rate dependency piezoresistive compression tests are shown in Figure 8a,b. Both sensors demonstrated some inconsistencies in the relative resistance change over 12 h of cyclic compressive loading, however, the least porous sponge demonstrated the more stable piezoresistive behavior. Additionally, the CNT3P70 sponge displayed more consistent piezoresistive sensitivity than the CNT3P85 sponge for significantly varying strain rates (Figure 8c,d). This result may be explained by the significantly larger energy absorbed and energy recovered in the least porous sponge compared to the most porous sponge as shown in the stress-strain curves in Figure 7c. Due to the differences in the viscoelastic responses, the CNT3P70 sponge displayed more reliable piezoresistive sensitivity regardless of strain rate, except for very small strains at 1000%/min strain rate. In contrast, the CNT3P85 sponge displayed more noticeable decreases in piezoresistive sensitivity as the strain rate increased. Overall, these additional piezoresistive tests showed that CNT3P70 sponges displayed the most dependable piezoresistive behavior, therefore, the material formulation was considered optimal and was used for subsequent sensor application demonstrations. 

### 3.4. Flexible Sensor Applications

**Figure 9:**
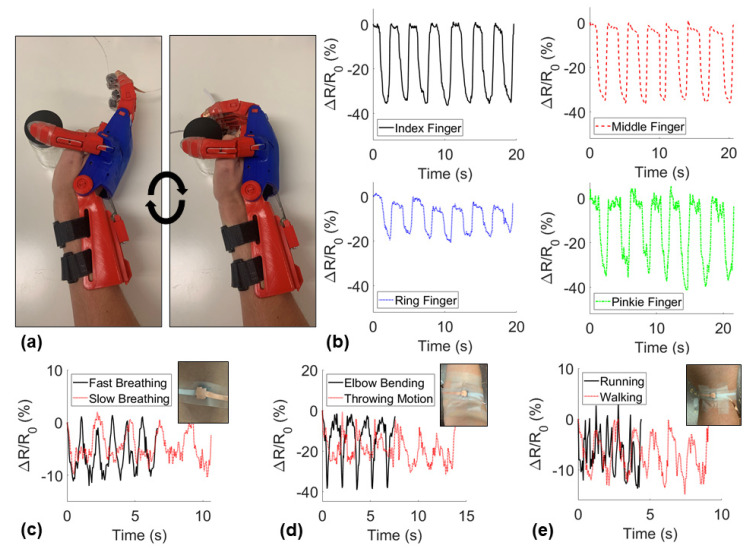
Demonstration of applications of sponge sensors including (**a**,**b**) fingertip pressure detection on a prosthetic hand cyclically grabbing an object, and skin-attachable human motion detection on the (**c**) chest for breath monitoring, (**d**) inside of the elbow, and (**e**) back of the knee during running and walking.

It is imperative to consider the health implications when developing any nanocomposite sensor that will be attached to or used by a person as CNTs and other nanoparticles may be detrimental to the health of humans. Many studies have fabricated highly sensitive piezoresistive sponges that have nanoparticles attached to the pore walls of a neat PDMS sponge. This common sensor manufacturing technique, when implemented for human motion detection, ignores the high likelihood that nanoparticles become detached from the surface of the elastomer due to repeated cycles and could be harmful to the health of the user, and therefore may not be appropriate for commercial human motion detection products. This study embedded the nanoparticles within the polymeric matrix to significantly reduce the likelihood of nanoparticle detachment during use. This advantage made the sensors in this study more applicable for skin attachable sensors and prosthetic sensors for dynamic and step motion detection. Piezoresistive signals of the sensors during the applications demonstrated are shown in Figure 9.

The piezoresistive responses of the sponges attached to each finger of a prosthetic hand are shown in Figure 9a where the minimum relative resistance measurements denoted grabbing an object and the maximum occurring after releasing the object. The sponges demonstrated consistent negative piezoresistive behavior during the step sensing application. Next, the sponges were attached to the skin of a user on the chest, behind the knee, and on the crease on the elbow to detect normal human motions. An individual’s breathing was measured before and after light exercise while the sensor was attached to the chest (Figure 9b). With the sensor attached behind the knee, the sensor detected both running and walking motions. The sensor attached to the elbow was able to produce distinctly different signals for different motions including a dynamic bending motion and a cyclic throwing motion. These demonstrations proved the potential for these nanocomposite sponge sensors to be used in a variety of motion-detecting applications. Lastly, a University of Oklahoma logo sponge, a gradient porosity sponge, and a triangular pyramid-shaped sponge were fabricated showing the diversity of sponges able to be fabricated with the method used in this study (Figure S4).

## 4. Conclusions

Piezoresistive sponges with tailorable electrical and mechanical properties were manufactured with a fabrication process that utilized a novel microwave-assisted rapid porogen removal method that significantly reduced the manufacturing time. The resistance and stiffness of the sponges were able to be varied by an order of magnitude by fabricating with various CNT loadings and sugar porogen amounts. The optimal CNT loading for the sensor was found to be 3 wt% to produce sponges with the highest conductivity and compressive strain sensitivity. The least porous sensor (CNT3P70) demonstrated the highest compressive strain sensitivity (between 5–50% strain) among the sensors with various porosities. The highly sensitive lower porosity sensor demonstrated higher piezoresistive consistency for a long-term cyclic durability test and a strain rate dependency test. The best sensor formulation was determined to be CNT3P70 and was demonstrated to be applicable as a skin-attachable sensor for dynamic human motion detection and step-sensing detection on a prosthetic hand.

## Figures and Tables

**Table 1:** Comparison of time required to remove the sugar porogen via microwave-assisted dissolution with different porogen removal methods used to fabricate PDMS sponges.

Material	Porogen	Removal Method	Time	Reference
PDMS/CNT	sugar	microwave irradiation in water	<5 min	this work
PDMS/CNT	sugar	boiling water and sonication	>24 h	[33]
PDMS/CNT	salt	immersed in heated water	72 h	[26]
PDMS/SrTiO_3_	salt	stirring in water	4 h	[27]
PDMS	polycaprolactone	sonication in acetone	1 h	[28]
PDMS	salt	dissolution in water	12–24 h	[34]
PDMS	polystyrene spheres	dissolved in acetone	24 h	[35]
PDMS	sugar	dissolution in water	24 h	[36,37,38]
PDMS	citric acid monohydrate	dissolved in ethanol	6 h	[39]
PDMS (graphene)	nickel	15 wt% HCL immersion	12 h	[40]
PDMS (graphene)	sugar	sonication in water	2 h	[24]
PDMS (graphene)	salt	dissolution in heated water	72 h	[41]
PDMS (nanodiamond)	sugar	dissolution in water	3 h	[42]
PDMS (CNTs)	sugar	sonication in hot water	1 h	[29]

**Table 2:** Comparison of gauge factor of the reported nanocomposite sensor with published sensor data in the literature.

Material	Gauge Factor	Applied Strain	Porosity	Reference
PDMS/CNT	6.20	10% compression	76%	this work
PDMS/carbon nanofiber (CNF)	6.50	40% tension	~79%	[20]
PDMS/CNF	1.65	10% compression	N/A	[44]
PDMS/Graphene	3.63	10% tension	N/A	[45]
PDMS/Graphene	8.77	9.5% compression	N/A	[21]
PDMS/Carbon Black	~2.0	10% compression	76.1%	[46]
PDMS/CNF	3.1	15% compression	74.7%	[22]
PDMS/CNT	7.9	10% compression	N/A	[17]

N/A: data not available in the references.

## Data Availability

The data presented in this study are available on request from the corresponding author.

## Supplementary Materials

The following are available online at https://www.mdpi.com/article/10.3390/nano11071740/s1, Figure S1: Comparison of the (a) compressive modulus and (b) the measured porosities of the CNT1.5P70, CNT2P70, CNT2.5P70, and CNT3P70 sponges, Figure S2: Piezoresistive step-sensing comparison of (a) the lowest porosity sponge (CNT3P70) and (b) the highest porosity sponge (CNT3P85) for 5%, 10%, and 25% compressive strains, Figure S3: Viscoelastic creep comparison of the piezoresistive and stress response for (a) the lowest porosity sponge (CNT3P70) and (b) the highest porosity sponge (CNT3P85) held at 50% compressive strain for 1 h, Figure S4: Pictures of fabricated nanocomposite sponges including (a) the University of Oklahoma logo, (b) a sample with gradient porosity, and (c) a triangular pyramid; Table S1: Designation of investigated sponge materials.
