# Supplementary material for: PDMS Sponges with Embedded Carbon Nanotubes as Piezoresistive Sensors for Human Motion Detection
Source: Nanomaterials (Basel). 2021 Jul 1;11(7):1740. doi: 10.3390/nano11071740 (PMC8308176; doi:10.3390/nano11071740)
Supplement: Supplementary file 1 [file nanomaterials-11-01740-s001.zip › nanomaterials-1261372-supplementary.pdf]

nanomaterials MDPI

Supplementary material

PDMS Sponges with Embedded Carbon Nanotubes as Piezoresistive Sensors for Human Motion Detection

Blake Herren, Vincent Webster, Eric Davidson, Mrinal C. Saha, M. Cengiz Altan and Yingtao Liu *

School of Aerospace and Mechanical Engineering, University of Oklahoma, Norman, OK 73019, USA; blake.herren@ou.edu (B.H.); Vincent.G.Webster-1@ou.edu (V.W.); ericdavidson@ou.edu (E.D.); msaha@ou.edu (M.C.S.); altan@ou.edu (M.C.A.)

* Correspondence: yingtao@ou.edu; Tel.: +1-405-325-3663

Table S1. Designation of investigated sponge materials.

Sponge Material Designation	Sugar Porogen Amount (wt%)	CNT Loading (wt%)
CNT1.5P70	70	1.5
CNT2P70	70	2
CNT2.5P70	70	2.5
CNT3P70	70	3
CNT3P75	75	3
CNT3P80	80	3
CNT3P85	85	3

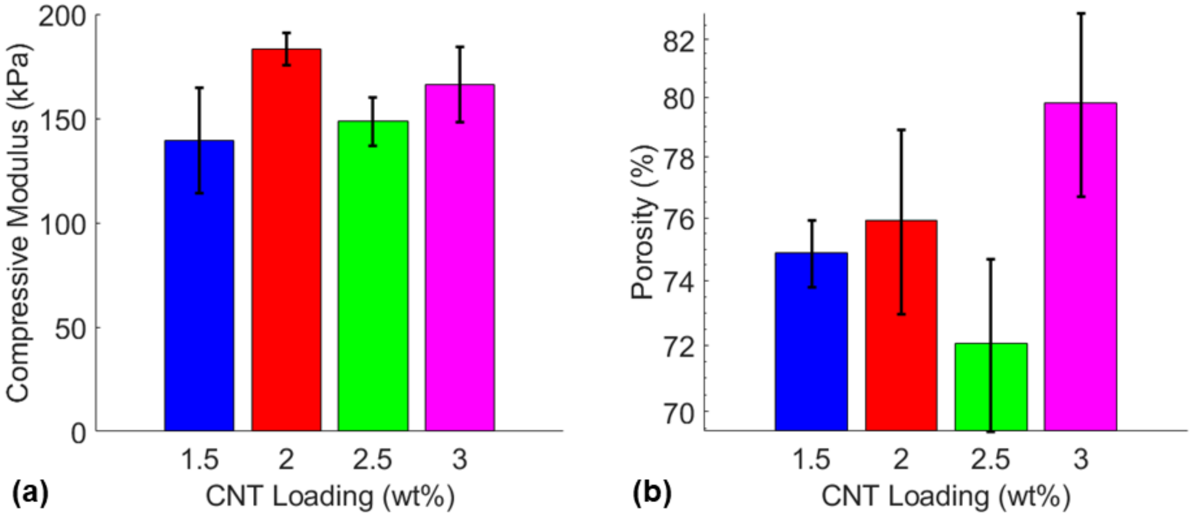

Figure S1. Comparison of the (**a**) compressive modulus and (**b**) the measured porosities of the CNT1.5P70, CNT2P70, CNT2.5P70, and CNT3P70 sponges.

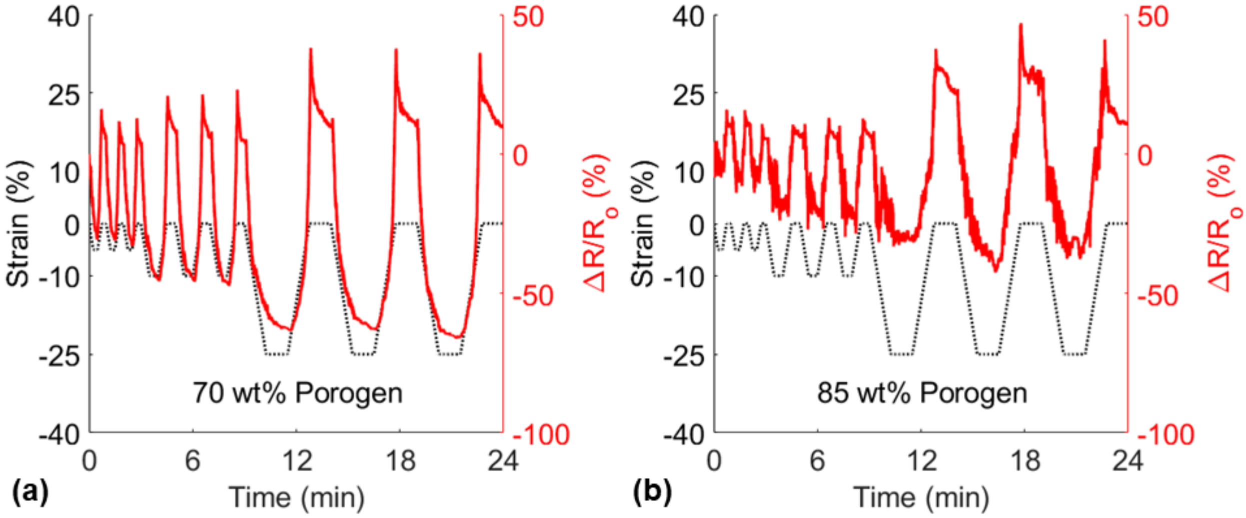

Figure S2. Piezoresistive step-sensing comparison of (**a**) the lowest porosity sponge (CNT3P70) and (**b**) the highest porosity sponge (CNT3P85) for 5 %, 10 %, and 25 % compressive strains.

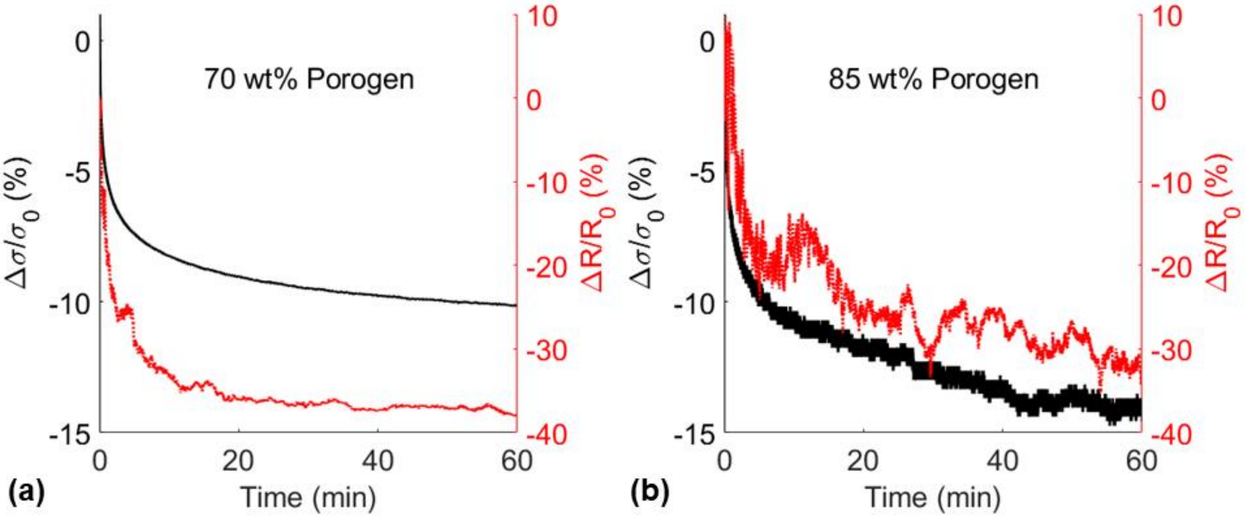

Figure S3. Viscoelastic creep comparison of the piezoresistive and stress response for (**a**) the lowest porosity sponge (CNT3P70) and (**b**) the highest porosity sponge (CNT3P85) held at 50 % compressive strain for 1 h.

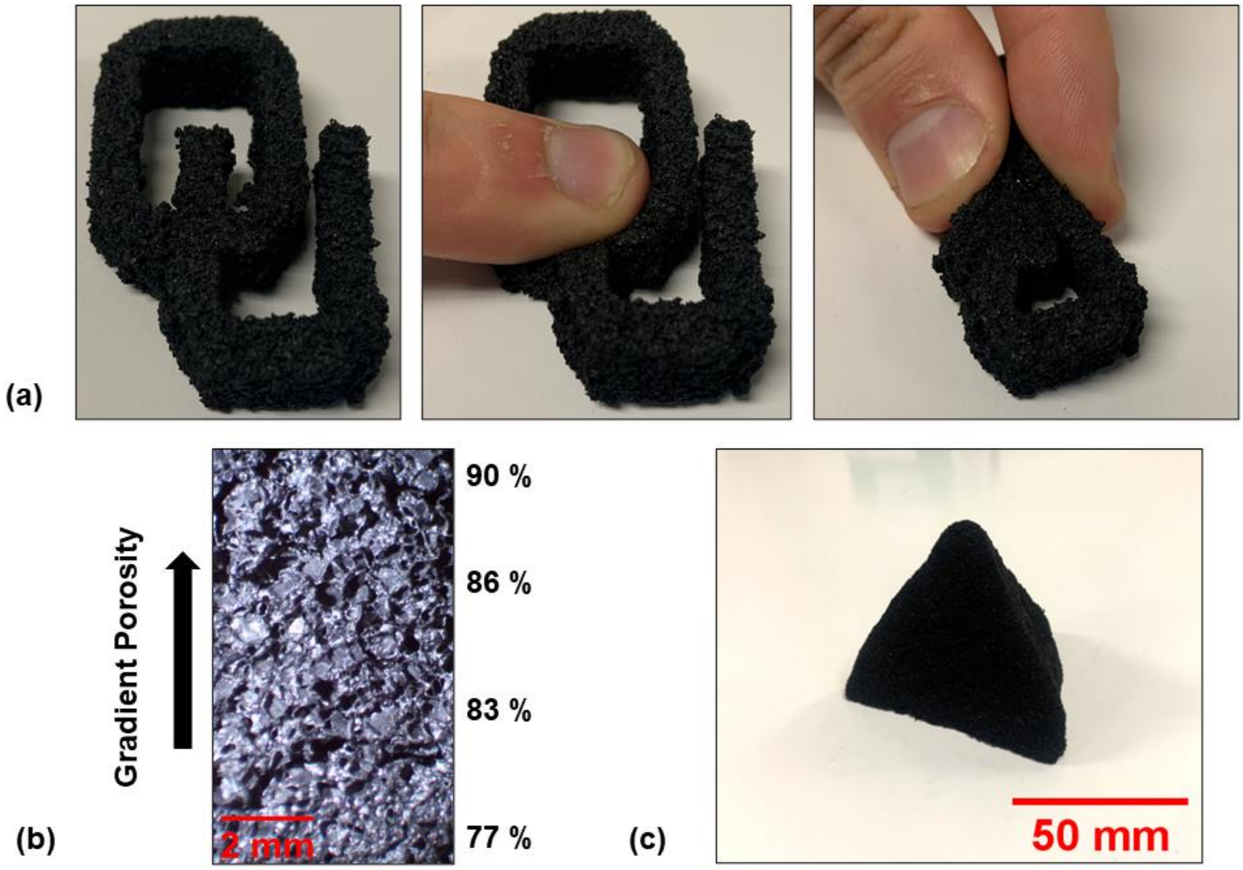

Figure S4. Pictures of fabricated nanocomposite sponges including (**a**) the University of Oklahoma logo, (**b**) a sample with gradient porosity, and (**c**) a triangular pyramid.
